# Phenolic metabolism in the hornwort *Anthoceros agrestis*: 4-coumarate CoA ligase and 4-hydroxybenzoate CoA ligase

**DOI:** 10.1007/s00299-020-02552-w

**Published:** 2020-05-13

**Authors:** Julia Wohl, Maike Petersen

**Affiliations:** Institut für Pharmazeutische Biologie Und Biotechnologie, Philipps-Universität Marburg, Robert-Koch-Str. 4, 35037 Marburg, Germany

**Keywords:** *Anthoceros agrestis*, Benzoic acids, Coenzyme A ligase, Hornworts, Hydroxycinnamic acids, Phenylpropanoid metabolism

## Abstract

**Key message:**

4-Coumarate coenzyme A ligase and 4-hydroxybenzoate coenzyme A ligase from the hornwort *Anthoceros agrestis* expressed in *E. coli* were characterized on biochemical and molecular levels and showed interesting substrate specificities.

**Abstract:**

Acyl-activating enzymes are associated with the biosynthesis or degradation of various metabolic products such as lipids, amino acids, sugars, and natural compounds. In this work, cDNA sequences encoding 4-coumarate coenzyme A ligase (4CL) and 4-hydroxybenzoate coenzyme A ligase (4HBCL) were amplified from the hornwort *Anthoceros agrestis*. The coding sequences were expressed in *E. coli* and purified by Ni-chelate chromatography. The CoA ligases exhibited different substrate specificities. 4CL catalyzed the activation of 4-coumaric acid, 3-coumaric acid, 2-coumaric acid, caffeic acid, isoferulic acid, ferulic acid, and cinnamic acid but lacked activities towards sinapic acid and benzoic acids. In contrast, 4HBCL preferred 4-hydroxybenzoic acid and benzoic acid, but also accepted other benzoic acid derivatives except salicylic acid and 3-aminosalicylic acid. Furthermore, 4HBCL also activated isoferulic acid, cinnamic acid, 2-coumaric acid, 3-coumaric acid, 4-coumaric acid and caffeic acid, but lacked affinity for ferulic acid and sinapic acid. These substrate specificities could be related to the phenolic compounds identified in *Anthoceros agrestis*.

**Electronic supplementary material:**

The online version of this article (10.1007/s00299-020-02552-w) contains supplementary material, which is available to authorized users.

## Introduction

The hornwort *Anthoceros agrestis* Paton (Anthocerotaceae) has recently been proposed as a model plant for investigations of developmental processes in hornworts and land colonization from the aquatic environment by plants (Szövényi et al. 2015) most probably from a member of the Zygnematophyceae (Wickett et al. 2014; Rensing 2018). The conquest of land posed a number of challenges to plants, amongst others the necessity to develop cell wall reinforcements and strategies against water loss as well as UV screens and defences against herbivores and pathogens. Various phenolic compounds deduced from the aromatic amino acids l-phenylalanine and—to a lesser extent—l-tyrosine are known to contribute to overcoming these challenges. In hornworts, phenolic metabolism is especially elaborated. More than 25 phenylpropanoid-derived specialized compounds have been described in phytochemical investigations of *Anthoceros agrestis* (Takeda et al. 1990; Trennheuser 1992; Trennheuser et al. 1994; Vogelsang et al. 2006). The most prominent phenolic compound is rosmarinic acid (RA), an ester of caffeic acid and 3-(3,4-dihydroxyphenyl)lactic acid which is especially well known as compound occurring in species of the Lamiaceae and Boraginaceae, but turning up as specialized metabolite sporadically throughout the plant kingdom (Petersen and Simmonds 2003; Petersen et al. 2009). The biosynthesis of RA has been fully elucidated in *Coleus blumei* (Lamiaceae) on molecular and biochemical levels (Petersen et al. 1993). One of our current interests is to show whether the ability to synthesize RA has evolved once or several times independently during evolution. For this purpose, the enzymes and genes of the phenylpropanoid pathway and RA biosynthesis in *Anthoceros agrestis* are under investigation.

Acyl-activating enzymes (AAEs) are responsible for the activation of carboxylic acids through a two-stage reaction (Shockey and Fulda 2003). In the first step, the substrate is activated by transfer of AMP from ATP under release of pyrophosphate. The resulting unstable substrate-AMP intermediate remains bound in the active center of the enzyme. In the second step, free electrons of the sulfur group of the acyl acceptor coenzyme A causes a nucleophilic attack on the carbon atom of the carboxyl group, thereby AMP is released and a thioester is formed. There are several AAEs that allow the activation of various substrates like acetate and fatty acids of different length (Watkins 1997), benzoic acid derivatives (Chang et al. 1997), cinnamic acid derivatives (Ehlting et al. 1999) or citrate, malate and malonate (An et al. 1999). In most cases, the acyl moiety is transferred to coenzyme A, but other acceptors are also described, for example, amino acids or molecular oxygen (Conti et al. 1996; Staswick and Tiryaki 2004).

The formation of 4-coumaroyl-CoA (4-hydroxycinnamoyl-CoA) is the last step in the core phenylpropanoid pathway and 4-coumaroyl-CoA is the precursor for many specialized metabolites. The reaction is catalyzed by 4-coumarate coenzyme A ligase (4CL; E.C. 6.2.1.12). In addition to 4-coumaric acid cinnamic, caffeic, ferulic, isoferulic and sinapic acids are often accepted. Nevertheless, phenylpropanoid CoA ligases differ substantially in substrate specificity and preference (Lindermayr et al. 2002). Schneider et al. (2003) identified 12 amino acids proposed to function as the 4CL substrate specificity code, two of these 12 amino acids were essential for acceptance of sinapic acid. In plants, the 4CL protein is found soluble in the cytosol. Usually several paralogs are found in plants and they mostly have different expression patterns (Renault et al. 2019). A phylogenetic analysis of almost 200 4CL sequences suggested that a duplication event had taken place in seed plants before the split of gymnosperms and angiosperms (Li et al. 2015). Homologs of 4CL are already present in the genomes of green algae, red algae, glaucophytes, diatoms, dinoflagellates, haptophytes, cryptophytes, and oomycetes (Labeeuw et al. 2015). Therefore, it is proposed that the gene set enabling the formation of phenylpropanoids had been evolved prior to the appearance of lignin as cell-wall reinforcing structure.

Benzoic acid derivatives can either be synthesized directly from intermediates of the shikimate pathway, mainly leading to salicylic acid and its derivatives, or from the phenylpropanoid pathway by β-oxidative chain shortening of cinnamic acid or 4-coumaric acid leading to benzoic acid (BA) or 4-hydroxybenzoic acid (4HBA), respectively. On the other hand, benzoic acids can also be produced by a non-oxidative pathway in the cytosol (Wildermuth 2006; Widhalm and Dudareva 2015). Probably the most prominent representative of the BA-derived products is salicylic acid (SA) and its volatile form methyl-SA (Leon et al. 1995; Ogawa et al. 2005; Ogawa et al. 2006; Pan et al. 2006; Sawada et al. 2006). SA is nowadays regarded as a plant hormone and signaling compound not only involved in local and systemic disease defense responses but also involved in other processes, e.g., thermoregulation (Vlot et al. 2009). Benzoic acid functions as a precursor for aromatic cytokinins (Werbrouck et al. 1996; Strnad 1997; Tarkowská et al. 2003; Mutui et al. 2012), aroma and flavor compounds (Wang and De Luca 2005; Goff and Klee 2006; Dudareva et al. 2006, 2013; Schwab et al. 2008) and plant defense metabolites (Dudareva et al. 2004; Qualley and Dudareva 2008; Köllner et al. 2010). 4-Hydroxybenzoic acid is supposed to be involved in the biosynthesis of ubiquinone (Gaisser and Heide 1996; Block et al. 2014; Tohge et al. 2014), although the exact pathway is yet unclear.

Benzoate coenzyme A ligases have rarely been described in plants. Bacterial benzoate CoA ligases (badA) function in the anaerobic degradation of diverse aromatic compounds (Gibson et al. 1994; Egland et al. 1995). In *Arabidopsis thaliana*, a benzoate CoA ligase (E.C. 6.2.1.25) is involved in the formation of benzoyloxyglucosinolates (Kliebenstein et al. 2007) and an *o*-succinylbenzoate CoA ligase (E.C. 6.2.1.26) in phylloquinone biosynthesis (Kim et al. 2008). Furthermore, benzoate CoA ligase is putatively involved in the biosynthesis of the floral scent compound benzylbenzoate in *Clarkia breweri* (Beuerle and Pichersky 2002). 3- and 4-Hydroxybenzoate CoA ligases (E.C. 6.2.1.37, 6.2.1.27) act in xanthone biosynthesis in *Centaurium erythraea* (Barillas and Beerhues 1997) and benzophenone biosynthesis in *Hypericum androsaemum* (Schmidt and Beerhues 1997). A cinnamoyl CoA ligase also forming benzoyl-CoA has been characterized from *Petunia hybrida* (Klempien et al. 2012).

In this work, we describe the functional expression of two coenzyme A ligases from the hornwort *Anthoceros agrestis*, both with specific substrate affinities. The first, a 4CL, only accepted hydroxycinnamic acids, preferably coumaric and isoferulic acid but not benzoic acid derivatives. The other CoA ligase preferably accepted benzoic acid or monohydroxylated benzoic acids (except salicylic acid) and a range of other benzoic acid derivatives as well as some (hydroxy)cinnamic acids. To our knowledge, this is the first report on a (4-hydroxy)benzoate coenzyme A ligase (4HBCL) in bryophytes and the first functional characterization of 4CL from hornworts.

## Materials and methods

### Plant cell cultures

*Anthoceros agrestis* cell suspension cultures were cultivated as described previously (Petersen 2003).

### Preparation of cDNA and amplification of partial sequences of Aa4CL and Aa4HBCL

RNA isolation was performed according to Chomczynski and Sacchi (1987). cDNA was prepared with the RevertAid™ First Strand cDNA Synthesis Kit (Fermentas) after checking RNA integrity. Internal PCR primers were designed based on *Anthoceros agrestis* scaffold 35,279 (Szövenyi, personal communication; Szövényi et al. 2015) and *Anthoceros punctatus* (a close relative to *A. agrestis*) scaffold 6378 (*Anthoceros punctatus* database: https://bioinformatics.plants.ox.ac.uk/anthoceros/blast.html). All primers were synthesized by Eurofins Genomics (for primer sequences see Suppl. Table S1). PCR assays of 25 µl were conducted with up to 0.2 µg cDNA, 0.5 µl 10 mM dNTP mix, 0.5 µl of each primer (Aa35279_f + Aa35279_r; AaAp6378_f + AaAp6378_r 100 µM), 3.0 µl 25 mM MgCl_2_, 5.0 µl 5 × GoTaq buffer and 0.1 µl GoTaq polymerase (5 U/µl, Promega) using the following program: 1 cycle 94 °C 120 s, 52–60 °C 60 s, 70 °C 90 s; 38 cycles 94 °C 30 s, 52–60 °C 60 s, 70 °C 90 s; 1 cycle 94 °C 120 s, 52–60 °C 60 s, 70 °C 600 s. Isolation of PCR products was done with the NucleoSpin Gel and PCR Clean-up Kit (Macherey–Nagel) after agarose gel electrophoresis. The sequence was determined (Seqlab) after ligation into pDrive (Qiagen), transformation and multiplication in *E. coli* EZ (Qiagen).

### RACE-PCR and amplification of full-length Aa4CL and Aa4HBCL sequences

According to the sequences determined in the previous step, RACE primers (Aa35279_5′R, Aa35279_3′R, AaAp6378_5′R, AaAp6378_3′R; Suppl. Table S1) were designed. RACE-PCR cDNA synthesis and PCR were performed using the SMARTer^®^ RACE 5′/3′ kit (Takara/Clontech). After the isolation of the PCR products (NucleoSpin Gel and PCR Clean-up Kit, Macherey–Nagel) and ligation into pDrive, *E. coli* EZ were transformed and grown overnight. The plasmid was isolated and the sequence determined (Seqlab). Two potential start codons were found for Aa4CL. For the amplification of the full-length sequences of Aa4CL and Aa4HBCL, primers with restriction sites were designed for XhoI (Aa35279) and PstI (AaAp6378) in the forward and HindIII in the reverse primer (Aa35279_fl_f, Aa35279_fl_r, AaAp6378_fl1_f, AaAp6378_fl2_f, AaAp6378_fl_r; Suppl. Table S1) for integration into the expression vector pRSET C (Invitrogen). PCRs of 25 µl were performed as above but with Phusion^®^ High-Fidelity DNA Polymerase (2 U/µl; NEB) and buffer (NEB) using the same PCR program as above, but with annealing temperatures of 58 °C for Aa35279 and 59 °C for AaAp6378. *E. coli* EZ cells were transformed after the ligation of the purified PCR products into pDrive. The Aa4HBCL (= Aa35279) full-length sequence (GenBank MN922306) and the two full-length sequences of Aa4CL (= AaAp6378) with alternative start codons (Aa4CL_1 and Aa4CL_2; GenBank MN922305) were integrated into the expression vector pRSET C. By integration, the sequences were attached to the sequence encoding an *N*-terminal 6xHis-tag already present on the expression vector pRSET C. The sequences were again checked for correctness (Seqlab) after transformation (*E. coli* EZ) and plasmid isolation.

### Protein expression, isolation, and purification

*E. coli* SoluBL21 (Amsbio) were transformed with the plasmids containing Aa4HBCL, Aa4CL_1 or Aa4CL_2. Plasmid uptake and correctness of the sequence were once again checked after plasmid isolation. SoluBL21 transformants were incubated in LB or TB media (Lessard 2013) containing 100 μg/ml ampicillin for 2–3 h at 37 °C 160 rpm up to an OD_600_ of 0.4–0.6. Then protein formation was induced with 1 mM isopropyl-β-d-galactopyranoside (IPTG) and the cultures were incubated overnight at 25 °C and 160 rpm. Cells were harvested by centrifugation (3000*g*, 4 °C, 5 min) and the pellet was resuspended in 4 ml buffer (0.1 M K_2_HPO_4_/KH_2_PO_4_ pH 8.0). The cells were disrupted by ultrasonication on ice (5 × 30 s, 100%, 0.3 cycles) after 30 min incubation with appr. 50 mg lysozyme. The crude protein extract was obtained by centrifugation (5000*g*, 4 °C, 20 min) and was further purified on Ni–NTA resin (Novagen) via the *N*-terminally attached 6xHis-tag to obtain highly enriched Aa4CL or Aa4HBCL protein. After adjusting the crude protein extract to 10 mM imidazole and 300 mM NaCl, 1 ml pre-equilibrated Ni–NTA resin was added and incubated on ice for 1 h. The resin was washed with 6 ml wash buffer (50 mM K_2_HPO_4_/KH_2_PO_4_ pH 8.0, 15 mM imidazole, 300 mM NaCl) and eluted with 3 ml elution buffer (50 mM K_2_HPO_4_/KH_2_PO_4_ pH 8.0, 250 mM imidazole, 300 mM NaCl). The elution fractions were desalted by gel filtration through PD-10 columns (GE Healthcare) into 0.1 M K_2_HPO_4_/KH_2_PO_4_ buffer pH 7.0. All protein concentrations were determined according to Bradford (1976) using bovine serum albumin (1 mg/ml) as a standard after verification of linearity between absorption and protein concentration with a calibration curve. The purified protein preparations were stored at − 80 °C.

### SDS-PAGE and Western blotting

Protein extracts were subjected to SDS-PAGE essentially according to Laemmli (1970). After SDS-PAGE, the gel was either stained with Coomassie Brilliant Blue R250 or Western blotting was performed basically as specified by Mahmood and Yang (2012), but applying the Towbin et al. (1979) buffer system. The expressed proteins were detected with mouse anti-6xHis-tag monoclonal antibodies (ThermoFisher, MA1-21315), followed by goat anti-mouse antibodies conjugated to alkaline phosphatase (Life Technologies, A16087) as secondary antibody. Staining was performed with nitro blue tetrazolium chloride (NBT)/5-bromo-4-chloro-3-indolyl-phosphate (BCIP) following the standard protocol on https://www.sysy.com/protocols/blot.php. In short, the alkaline phosphatase coupled to the secondary antibody dephosphorylates BCIP and the resulting indoxyl dimerizes after oxidation by NBT resulting in a dark blue color (Sambrook and Russell 2001).

### Standard assays for coenzyme A activation of (hydroxy)cinnamic acid derivatives

Formation of CoA-esters from (hydroxy)cinnamic acid derivatives was determined photometrically at the absorption maxima published by Stöckigt and Zenk (1975) and Zenk (1979). A standard assay consisted of 100 mM K_2_HPO_4_/KH_2_PO_4_ pH 7.0, 2 µg (Aa4CL) or 32 µg (Aa4HBCL) purified protein, 1 mM dithiothreitol (DTT), 2.5 mM ATP, 2.5 mM MgCl_2_ and 500 µM (hydroxy)cinnamic acid. While testing caffeic acid as substrate, DTT was omitted, because the spectrophotometric assay was disturbed by DTT. The assay was incubated at 40 °C for 1 min and then the reaction was started by addition of 500 µM CoA and incubated for 8 min; absorption was recorded throughout the incubation time. The specific activity was calculated using the measured slope of the absorbance and the respective extinction coefficient published by Stöckigt and Zenk (1975) and Zenk (1979) for the (hydroxy)cinnamoyl-CoA thioesters. Data for kinetic analyses for Aa4CL were obtained from three independent protein isolations with at least three technical replicates for each substrate concentration. The results were analyzed with the GraphPad Prism 5 software using Michaelis–Menten, Lineweaver–Burk (not shown) and Hanes–Woolf models.

### Standard assays for coenzyme A activation of (hydroxy)benzoic acid derivatives

#### Direct assay

The activation of 4-hydroxybenzoic acid was measured photometrically at 300 nm. A standard assay for Aa4HBCL consisted of 100 mM K_2_HPO_4_/KH_2_PO_4_ pH 7.0, 15 µg purified protein, 1 mM DTT, 2.5 mM ATP, 2.5 mM MgCl_2_, and 500 µM 4-hydroxybenzoic acid. The assay was incubated at 45 °C for 1 min and then the reaction was started by the addition of 500 µM CoA and incubated for 8 min; absorption was recorded throughout the incubation time. The specific activity was calculated with the extinction coefficient for 4-hydroxybenzoyl-CoA of 13 cm^2^/µmol (Biegert et al. 1993). Data for kinetic values were measured using purified enzyme from three independent protein isolations with three technical replicates for each substrate concentration. The results were analyzed with the GraphPad Prism 5 software using Michaelis–Menten, Lineweaver–Burk (not shown) and Hanes–Woolf models.

#### Indirect assay

For the determination of CoA ligase activities with all other (hydroxy)benzoic acid derivatives, we established an indirect spectrophotometric assay using hexokinase (Sigma) and glucose-6-phosphate dehydrogenase (G6PDH, Fluka) to determine the remaining ATP concentration after incubation by measuring the conversion of NADP to NADPH at 340 nm in a two-step assay. The CoA ligase assay consisted of 250 µl 100 mM Tris–HCl, pH 7.5, 14 µg purified enzyme, 1 mM DTT, 500 µM ATP, 500 µM MgCl_2_, 500 µM (hydroxy)benzoic acid derivative, and 500 µM CoA. After incubation for 1 h at 40 °C, the reaction was stopped by heating for 5 min at 95 °C and cooling on ice. 750 µl reaction mixture consisting of 100 mM Tris–HCl, pH 7.5, 200 µM NADP, 200 µM glucose, 1 U hexokinase, and 1 U G6PDH was added and the absorption at 340 nm recorded for 20 min at 25 °C. In principle, the addition of glucose and hexokinase results in the ATP-dependent formation of glucose-6-phosphate which is oxidized to 6-phosphogluconolactone by G6PDH under reduction of NADP to NADPH which is measured at 340 nm. Kinetic values could not be determined with this method, as a difference in the ATP concentration added in saturating concentrations was not measurable after 3-min reaction time which was used to ensure the determination of initial reaction velocities.

### LC–MS analysis

Conversion of (hydroxy)benzoic acids was additionally analyzed by LC–MS. Assays were performed using 100 µl reaction volume consisting of 100 mM K_2_HPO_4_/KH_2_PO_4_, pH 7.0, 7.5 µg (Aa4CL) or 28 µg (Aa4HBCL) purified enzyme, 1.25 mM ATP, 1.25 mM MgCl_2_, 500 µM (hydroxy)benzoic acid derivative and 1 mM CoA. Assays were incubated at 40 °C for 30 min (Aa4HBCL, Aa4CL with 2-coumaric and 3-coumaric acid) or 60 min at 35 °C (Aa4CL with hydroxybenzoic acids). An assay with heat-denatured protein (10 min at 95 °C) served as negative control. Assays were stopped on ice by the addition of 100 µl methanol. After centrifugation for 10 min at 15,000*g*, 15 µl of the supernatant was analyzed by LC–MS. LC was performed on a HPLC 1260 (Agilent Technologies) with a Multospher 120 RP18 column (250 × 2 mm; particle size 5 μm) using a solvent system of A = 0.1% (v/v) aqueous formic acid, B = acetonitrile with 0.1% (v/v) formic acid at a flow rate of 0.5 ml/min and a temperature of 25 °C with the following gradient: 0–10 min 5% B → 100% B; 10–15 min 100% B; 15–15.1 min 100% B → 5% B; 15.1–20 min 5% B. Detection was performed with the mass spectrometer micrOTOF-Q III with ESI source (Bruker Daltonics) calibrated with 5 mM sodium formate using the negative mode.

## Results

### Isolation of cDNAs encoding 4CL and 4HBCL from *Anthoceros agrestis*

A nucleotide sequence of a putative 4CL (*Anthoceros punctatus* scaffold 6378; Kelly, personal communcation) has been identified by nucleotide BLAST search using a 4CL sequence from *Melissa officinalis* (FN665699.1) in the *Anthoceros punctatus* database (https://bioinformatics.plants.ox.ac.uk/anthoceros/blast.html). A partial sequence with primers directed against the *A. punctatus* sequence was amplified using cDNA from *A. agrestis*. After RACE-PCR, we obtained two potential Aa4CL full-length sequences due to two possible start codons. The open reading frames of the two sequences consisted of 1647 bp (Aa4CL_1) and 1599 bp (Aa4CL_2), encoding proteins of 548 amino acid residues (58.42 kDa) and 532 amino acid residues (56.86 kDa), respectively. The Aa4CL nucleotide sequence was deposited in GenBank under the accession number MN922305. The longer as well as the shorter amino acid sequence showed the highest identity (BLASTp) with 64% to a 4CL from the gymnosperm *Cryptomeria japonica* var. *sinensis* (QDC33553.1) which, however, has not been characterized biochemically.

On the search for further 4CL candidates, *Anthoceros agrestis* scaffolds 19917, 20832 and 35279 (Szövenyi, personal communication; Szövényi et al. 2015) were identified. Aa19917 seemed to be not expressed since amplification of a partial sequence with different primer combinations worked only using genomic DNA but not with cDNA (data not shown). The encoded protein of Aa20832 was synthesized in *E. coli*, but demonstrated no activity towards different cinnamic or benzoic acid derivatives and was thus not further characterized (data not shown).

The encoded protein of Aa35279 was later revealed to be active as a (4-hydroxy)benzoate CoA ligase (4HBCL; GenBank MN922306). After PCR amplification with primers directed against a partial sequence of 798 bp, RACE-PCR was performed to obtain the 5′- and 3′-ends of the cDNA. The full open reading frame of Aa4HBCL consisted of 1704 bp and could be translated into a protein of 567 amino acid residues with a molecular mass of 61.84 kDa. With an identity of 60%, the amino acid sequence was closest to 4-coumarate CoA ligase-like 1 from *Selaginella moellendorfii* (XP_002981856.1) in a BLASTp search.

**Fig. 1:**
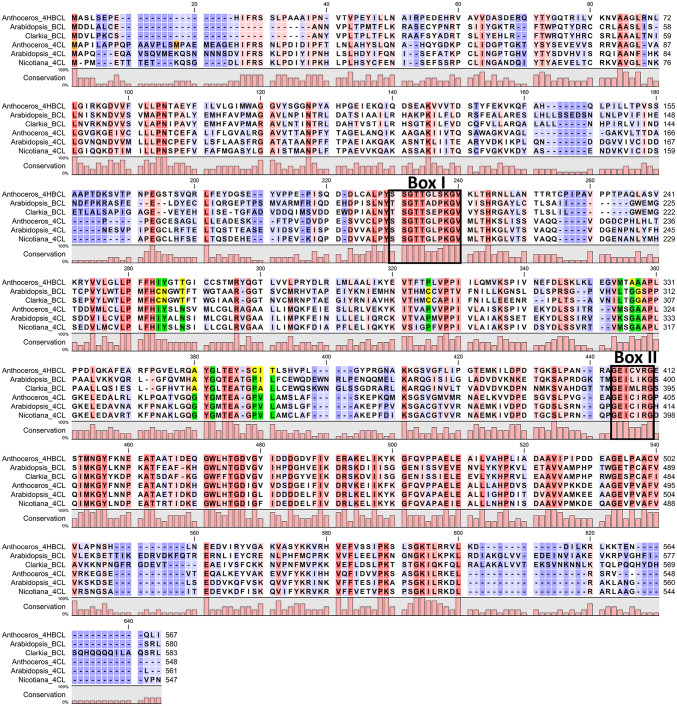
Alignment of amino acid sequences of different CoA ligases (4HBCL = 4-hydroxybenzoate CoA ligase, BCL = benzoate CoA ligase, 4CL = 4-coumarate CoA ligase) from *Anthoceros agrestis* (4CL: MN922305 and 4HBCL: MN922306), *Arabidopsis thaliana* (Q9SS01), *Clarkia breweri* (AEO52695.1), *Arabidopsis thaliana* (Q42524) and *Nicotiana tabacum* (O24145). Highly conserved amino acids are marked red, low conservation is marked blue. Typical conserved sequence motifs are marked by boxes. Amino acids from the 4CL substrate specificity code (Schneider et al. 2003) are highlighted green, and amino acids differing from this code are highlighted yellow. The two possible start methionine residues of Aa4CL are highlighted orange (color figure online)

Alignment of the two newly identified CoA ligase amino acid sequences from *Anthoceros agrestis* (EMBOSS Needle) resulted in an identity of 39.3% and a similarity of 58.5% to each other. Furthermore, the two CoA ligases from *Anthoceros agrestis* were aligned with different plant 4CLs and the two already characterized CoA ligases with benzoate-activating properties from *Arabidopsis thaliana* (Q9SS01) and *Clarkia breweri* (AEO52695.1) (Fig. 1). The alignment shows that the two motifs box I ([STG][STAG]G[ST][STEI][SG]X[PASLIVM][KR]) and box II (GEICIRG), corresponding to the core motifs A3 and A6 from the phenylalanine‐activating subunit (PheA) of gramicidin synthetase 1 (Conti et al. 1997; Stuible and Kombrink 2001), are highly conserved throughout the 4CL sequences and Aa4HBCL. In the two benzoate-activating enzymes, at least two amino acids in the core of both boxes differed. Schneider et al. (2003) identified 12 amino acids proposed to function as the 4CL substrate specificity code. These amino acids were all conserved in the characterized 4CLs and Aa4CL (Fig. 1, marked in green). In the benzoate-activating enzymes and Aa4HBCL, however, some of these amino acids differed (marked in yellow in Fig. 1). Three changes were particularly noticeable: N288T, G380A and V390I or V390A.

### Expression of Aa4CL in *E. coli* and characterization of Aa4CL

Aa4CL protein with an *N*-terminally attached 6xHis tag was synthesized in *E. coli* SoluBL21. After a first proof of enzyme activity using crude protein extract, the protein was purified by metal chelate chromatography to afford nearly pure protein that was identified by SDS-PAGE followed by Western blotting (Suppl. Fig. S1). Catalytic activity of the expressed protein could be detected for the substrates caffeic, 2-coumaric, 3-coumaric, 4-coumaric, cinnamic, ferulic and isoferulic acid. While there was a clear increase in the absorption for Aa4CL, no absorption change was noticed in the empty vector control. Sinapic acid as well as different benzoic acid derivatives (benzoic, salicylic, 4-hydroxybenzoic, 2,3-dihydroxybenzoic, 2,4-dihydroxybenzoic and protocatechuic acid) were not accepted (Suppl. Figs. S2, S3 and S4). The latter were assessed by LC–MS analyses and caffeic acid was used as a positive control. For the produced CoA esters analyzed by LC–MS, we observed the [M–H] pseudo molecular ion as well as the doubly charged molecular ion [M/2]–H, as already reported by Beuerle and Pichersky (2002) for benzoyl-CoA.

To ascertain the pH as well as the temperature optima of the enzymes, additional assays using ferulic acid were performed showing a pH optimum between pH 7.0 and 7.5 in a 100 mM potassium phosphate buffer and a temperature optimum at 40 °C (Suppl. Fig. S5).

Kinetic data for Aa4CL_1 and Aa4CL_2 were determined for 4-coumaric acid, caffeic acid, ferulic acid, isoferulic acid, and cinnamic acid as hydroxycinnamic acid substrates. Although Aa4CL_2 demonstrated higher specific activities (around twice) compared to the longer variant Aa4CL_1, the substrate affinities were the same (Table 1). For both enzyme variants, 4-coumaric acid and isoferulic acid were the substrates accepted with the highest affinity, followed by caffeic acid, ferulic acid, and cinnamic acid (Suppl. Fig. S6).

**Table 1:** Kinetic data for Aa4CL_1 (a) and Aa4CL_2 (b)

	*K*_m_ (µM)	*V*_max_ (mkat/kg)	*K*_cat_ (1/s)	*k*_cat_/*K*_m_ (1/s mM)
(a) Aa4CL_1				
Cinnamic acid	218.2 ± 74.7	16.0 ± 2.9	1.0 ± 0.2	4.6
4-Coumaric acid	12.8 ± 4.0	24.0 ± 3.1	1.5 ± 0.2	118.5
Caffeic acid	15.5 ± 1.9	16.8 ± 2.2	1.1 ± 0.1	68.3
Ferulic acid	61.4 ± 21.9	25.3 ± 3.9	1.6 ± 0.2	26.1
Isoferulic acid	9.9 ± 1.8	13.1 ± 0.3	0.8 ± 0.02	84.1
ATP	227.1 ± 31.8	18.1 ± 4.8	1.1 ± 0.3	5.0
CoA	10.2 ± 0.2	15.9 ± 4.6	1.0 ± 0.3	98.5
(b) Aa4CL_2				
Cinnamic acid	216.7 ± 86.1	28.5 ± 2.9	1.8 ± 0.2	8.1
4-Coumaric acid	11.8 ± 4.4	41.2 ± 4.8	2.5 ± 0.3	215.1
Caffeic acid	20.5 ± 4.6	32.3 ± 5.8	2.0 ± 0.4	97.4
Ferulic acid	65.3 ± 21.9	49.4 ± 8.1	3.1 ± 0.5	46.7
Isoferulic acid	7.5 ± 1.8	28.0 ± 3.9	1.7 ± 0.2	231.6
ATP	151.1 ± 25.3	31.2 ± 1.0	1.9 ± 0.1	12.7
CoA	14.6 ± 3.2	35.8 ± 2.2	2.2 ± 0.1	151.6

*K*_m_ and *V*_max_ were calculated from Michaelis Menten plots by the GraphPad Prism software. Data were obtained from three technical replicates for each of the three biological replicates. All values represent mean ± standard error

For the co-substrate ATP Aa4CL_1 displayed a *K*_m_ value of 227.1 ± 31.8 µM (*V*_max_ 18.1 ± 4.8 mkat/kg) and Aa4CL_2 of 151.1 ± 25.3 µM (*V*_max_ 31.2 ± 1.0 mkat/kg) (Table 1; Suppl. Fig. S6). The *K*_m_ value for CoA was at 10.2 ± 0.2 µM (*V*_max_ 15.9 ± 4.6 mkat/kg) for Aa4CL_1 and 14.6 ± 3.2 µM (*V*_max_ 35.9 ± 2.2 mkat/kg) for Aa4CL_2 (Table 1; Suppl. Fig. S6).

### Expression of Aa4HBCL in *E. coli* and characterization of Aa4HBCL

Aa4HBCL (Genbank MN922306) carried an *N*-terminally attached 6xHis tag by insertion into the expression vector pRSET C and was also expressed in *E. coli* SoluBL21. At first, crude protein extracts of two different transformants, expressed either in TB or LB media, were analyzed by SDS-PAGE and Western blot analysis. All transformants showed a band of the expected size (~ 65 kDa) and protein formation was slightly higher using TB media compared to LB (Suppl. Fig. S7a). The protein was purified by metal chelate chromatography (Suppl. Fig. S7b) and tested for substrate acceptance. Photometrical analysis of assays incubated with different (hydroxy)cinnamic acids or 4-hydroxybenzoic acid (4HBA) resulted in an activation of 4HBA, isoferulic, cinnamic, 4-coumaric and caffeic acids; ferulic and sinapic acid were not converted (Suppl. Fig. S8). Crude protein extract from bacteria transformed with the empty vector served as negative control and showed no conversion of the applied substrates.

The activation of other benzoic acid derivatives was measured first with the indirect method. While a clear reduction of absorbance (based on a reduced concentration of ATP) could be detected for benzoic acid (BA) and 3-hydroxybenzoic acid (3HBA), this method was not sensitive enough to detect the conversion of the other tested substrates (Suppl. Fig. 9a). The attempt to determine kinetic values for BA, 3HBA, and 4HBA, using the indirect method, failed, because a reduction in the ATP concentration (applied at saturating concentrations for kinetic assays) was not detectable after 3-min reaction time, which was used to ensure the determination of initial reaction velocities. For this reason, only the relative conversion rates of the three substrates were compared (Suppl. Fig. S9b). The conversion rates of BA and 4HBA were the same after 30 min, with 68% (BA) and 67% (4HBA) remaining ATP after 30 min. 3HBA had the lowest conversion with 86% ATP remaining after 30 min.

The reaction products of all benzoic acid derivatives were also analyzed by LC–MS after incubation with higher protein amounts and longer incubation times (Suppl. Fig. S10). Here the formation of the expected CoA-ester in combination with the increase in AMP concentration was determined against a negative control (heat-denatured protein) after incubation for 30 min. The enzyme accepted BA, 3HBA, 4HBA, 2-aminobenzoic, 3-aminobenzoic, 2,3-dihydroxybenzoic, 2,4-dihydroxybenzoic, 3,4-dihydroxybenzoic (protocatechuic acid), and 2-amino-3-hydroxybenzoic acids. Moreover, the activation of 2-coumaric acid, 3-coumaric acid, 4-coumaric acid, and caffeic acid was verified by LC–MS. Salicylic acid (2HBA), 3-aminosalicylic acid (2-hydroxy-3-aminobenzoic acid), and vanillic acid (3-methoxy-4-hydroxybenzoic acid) were not converted; instead only the formation of AMP was observed. The same could be seen by applying glycolic acid as substrate (data not shown). For all produced CoA-esters, we observed the [M–H] pseudo-molecular ion as well as the doubly charged molecular ion [M/2]–H, as already reported by Beuerle and Pichersky (2002).

**Fig. 2:**
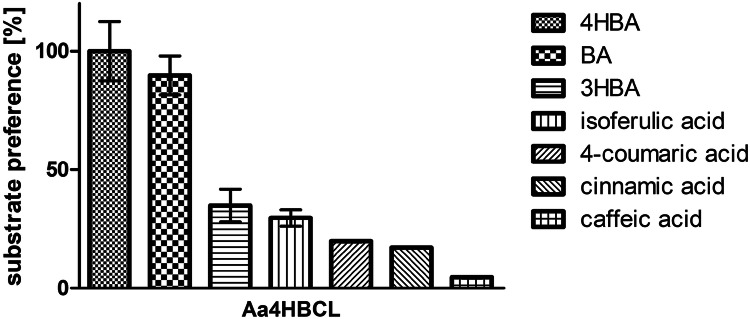
Substrate preference of Aa4HBCL in % compared to 4HBA. Data were collected either by the indirect assay for 0, 5, 15 and 30 min (4HBA, BA and 3HBA) or by direct photometerical measurement (4HBA, cinnamic acid, 4-coumaric acid, caffeic acid and isoferulic acid). Each bar represents the mean average of three technical replicates, the error bars represent the standard deviation

The relative activities for the accepted hydroxycinnamic acids and the three best accepted benzoic acids were compared to 4HBA (Fig. 2). With 90 ± 8%, BA was accepted almost as good as 4HBA. 3HBA and isoferulic acid were converted with a relative activity of 35 ± 7% and 30 ± 3%, respectively. The least converted substrates were 4-coumaric acid (20 ± 0.4%), cinnamic acid (17 ± 0.3%), and caffeic acid (5 ± 0.3%).

The formation of 4HBA-CoA could be directly determined spectrophotometrically at 300 nm (Biegert et al. 1993). First, the pH as well as the temperature optimum were determined. The temperature optimum was at appr. 50 °C. The pH optimum was at pH 7.3 in the assay when using a 0.1 M potassium phosphate buffer with pH 7.5 (Suppl. Fig. S11).

The kinetic values for 4HBA, CoA, and ATP were determined with the direct photometric method. The apparent *K*_m_ value for 4HBA was at 664.2 ± 1.5 µM and *V*_max_ at 16.5 ± 0.7 mkat/kg. The *K*_m_ values for ATP and CoA were at 1.2 ± 0.1 mM and 247.8 ± 19.9 µM, respectively, with *V*_max_ values of 23.4 ± 4.7 mkat/kg and 32.5 ± 0.8 mkat/kg (Table 2; Suppl. Fig. S12).

**Table 2:** Kinetic data for Aa4HBCL for its substrates 4HBA, CoA and ATP

	Aa4HBCL			
	*K*_m_ (µM)	*V*_max_ (mkat/kg)	*K*_cat_ (1/s)	*k*_cat_/*K*_m_ (1/s mM)
4HBA	664.2 ± 1.5	16.5 ± 0.7	1.1 ± 0.1	1.7
ATP	1175.3 ± 135.2	23.4 ± 4.7	1.6 ± 0.3	1.3
CoA	247.8 ± 19.9	32.5 ± 0.8	2.2 ± 0.1	8.7

*K*_m_ and *V*_max_ were calculated from Michaelis–Menten plots by the GraphPad Prism software. Data were obtained from three technical replicates for each of the three biological replicates. All values represent mean ± standard error

## Discussion

*Anthoceros agrestis* can accumulate RA up to 5% of the dry weight (Vogelsang et al. 2006). Other RA-related phenolic compounds as well as (hydroxy)cinnamic acid esters and amides have been identified as well (Takeda et al. 1990; Trennheuser 1992). Although the biosynthetic pathway for RA in the hornwort is not yet fully resolved, it is likely that activated hydroxycinnamic acids play a role. Thus, the presence of a 4CL was expected. Four potential *A. agrestis* 4CL genes were identified as scaffolds in databases from *A. agrestis* and a close relative, *A. punctatus*, the cDNAs amplified from *A. agrestis* and heterologously expressed in *E. coli*. Two of the isolated proteins (Aa4CL and Aa4HBCL) showed the formation of CoA-thioesters with phenolic acids. The third (Aa20832) was also heterologously expressed in *E. coli*, but until now its substrate(s) could not be identified although the reduction of ATP to AMP could be observed and thus the enzyme was considered to be active. The last potential partial sequence (Aa19917) could only be amplified with gDNA and not with cDNA in various attempts using different primer pairs, and we assume that this gene is presumably silenced.

The full-length sequence of Aa4CL had two possible start codons and both versions of the protein have been expressed and characterized (Aa4CL_1 and Aa4CL_2). We suppose that the second start codon is used for translation based on sequence alignments with other 4CLs and a higher specific activity of the expressed protein (Aa4CL_2). The two different *N* termini of the amino acid sequences could not be identified as a signal sequence (identification with SignalIP 4.1) and the nine nucleotides between the two possible start codons could not be attributed to a ribosome binding site (Kozak sequence). The alignment of 4CL sequences from *Anthoceros* and angiosperms (Fig. 1) demonstrated that the amino acids from box I and II (Uhlmann and Ebel 1993; Ehlting et al. 2001) are highly conserved throughout the plant kingdom and the 12 amino acids, which are supposed to act as the 4CL substrate specificity code, were always identical (Schneider et al. 2003). According to Schneider et al. (2003), the pattern of the last 2 of these 12 amino acids is important for the activation of sinapic acid. If both amino acids, valin and leucin, are present, sinapic acid is not converted, but if one of these amino acids is deleted, the enzyme acquires the new function towards sinapic acid (Lindermayr et al. 2002; Schneider et al. 2003). In Aa4CL, both amino acids were present and, therefore, it was not surprising that the enzyme showed no activation of sinapic acid. Nevertheless, other hydroxycinnamic acids were converted (Suppl. Figs. S2 and S3). Both expressed Aa4CL variants showed the same substrate affinities towards (hydroxy)cinnamic acids, whereas the formation of benzoyl-CoA derivatives could not be detected for Aa4CL (Suppl. Fig. S4).

Aa4CL2 (which we regard as the protein occurring in vivo; see above) accepts (hydroxy)cinnamic acids in the following order of affinities (*K*_m_): isoferulic > 4-coumaric > caffeic > ferulic > cinnamic acid (Table 1). The catalytic efficiencies (*K*_cat_/*K*_m_; Table 1) also reflect this order. However, the affinities and efficiencies for isoferulic acid and 4-coumaric acid are very close to each other. For comparison, kinetic data for 4CL with isoferulic acid as substrate are only available for *Petroselinum crispum* (26 µM; Knobloch and Hahlbrock 1977) and *Glycine max* (100 µM for isoenzyme 1 and 150 µM for isoenzyme 2; Knobloch and Hahlbrock 1975). This shows that Aa4CL has a considerably higher affinity towards isoferulic acid. The affinity for the second best substrate, 4-coumaric acid, with 11.8 ± 4.4 µM was in the range between 11 µM for 4CL from *Pinus taeda* (Chen et al. 2014) and 16 µM for *Physcomitrella patens* 4CL2 (Silber et al. 2008). For caffeic acid, the *K*_m_ values of different 4CLs ranged from 1 µM (*Nicotiana tabacum*; Li and Nair 2015) to 725 µM (*Physcomitrella patens* 4CL3; Silber et al. 2008). With 20.5 ± 4.6 µM Aa4CL_2 from *Anthoceros* was within this range. This was also the case for ferulic acid (65.3 ± 21.9 µM) with *K*_m_ values ranging from 2.2 µM (*Oryza sativa* 4CL3; Gui et al. 2011) to 800 µM (*Physcomitrella patens* 4CL3; Silber et al. 2008). Cinnamic acid is the substrate with the highest *K*_m_ value of 216.7 ± 86.1 µM for Aa4CL_2. This value is also in the range of published *K*_m_ values for *t*-cinnamic acid of 9.4 µM (*Oryza sativa* 4CL1; Gui et al. 2011) to 6.63 mM (*Arabidopsis thaliana*; Ehlting et al. 1999). With 151.1 ± 25.3 µM, the *K*_m_ value of Aa4CL_2 for ATP was comparably high; also in wild type and mutant enzymes of *Arabidopsis thaliana*, values ranged between 151 µM and 1.50 mM (Stuible et al. 2000). In *Forsythia suspensa* and *Glycine max,* the *K*_m_ value for CoA ranged between 3.2 µM and 7 µM (Gross and Zenk 1974; Knobloch and Hahlbrock 1975). This value was slightly higher for Aa4CL_2 with 14.6 ± 3.2 µM.

Trennheuser (1992) meticulously analyzed compounds isolated from suspension-cultured cells of *Anthoceros agrestis*. Among the identified (hydroxy)cinnamic acid derivatives were various esters and amides of 4-coumaric, caffeic and isoferulic acids while similar adducts with ferulic acid were missing. This is reflected in the substrate acceptance of Aa4CL with a higher affinity for isoferulic acid (*K*_m_ 7.5 µM) compared to ferulic acid (*K*_m_ 65.3 µM). This might be a specific feature of lower plants or hornworts since isoferulic acid is not a predominant compound in seed plants where ferulic acid and its derivatives are more important, e.g., as coniferyl alcohol unit (G-unit) in lignin formation.

The presence of a benzoic acid/hydroxybenzoic acid activating enzyme in the hornwort *Anthoceros agrestis* was less expected. Bacterial and fungal benzoate CoA ligases function in the catabolic pathway of aromatic carboxylic acids (Thornburg et al. 2015). In *Arabidopsis thaliana*, benzoate CoA ligase is involved in glucosinolate biosynthesis (Kliebenstein et al. 2007) and a 3-hydroxybenzoate coenzyme A ligase from *Centaurium erythraea* acts in xanthone biosynthesis (Barillas and Beerhues 1997). Moreover, the production of taxanes in *Taxus cuspidata* is dependent on benzoyl-CoA (Walker and Croteau 2000). The role of Aa4HBCL in the hornwort is not yet known; however, hydroxybenzoic, protocatechuic, and vanillic acid conjugates have been identified by Trennheuser (1992) in *Anthoceros agrestis*. An alignment of the two published plant (hydroxy)benzoate CoA ligases from *Arabidopsis thaliana* (Q9SS01) and *Clarkia breweri* (AEO52695.1) with Aa4HBCL and Aa4CL (Fig. 1) revealed that some amino acids of the 4CL substrate specificity code might also be important for the acceptance of benzoic acid derivatives. However, all three proteins had a threonine residue in position 288 instead of an asparagine. Moreover, position 380 was changed from a glycine to an alanine residue. In position 390, valine was changed either to isoleucine or alanine.

Aa4HBCL preferably activated 4-hydroxybenzoic acid, benzoic acid and 3-hydroxybenzoic acid, but also accepted other benzoic acid derivatives at a lower rate (Suppl. Figs. S9 and S10). Salicylic acid and 3-aminosalicylic acid were not converted. The same substrate preference, but different affinities were found in 3-hydroxybenzoate CoA ligase from *Centaurium erythraea* (Barillas and Beerhues 1997). While this enzyme preferred 3HBA and converted BA and 4HBA at much lower (but similar) rates, Aa4HBCL not only favored 4HBA and BA, but also accepted 3HBA with a considerably lower conversion rate. In addition, Aa4HBCL also converted cinnamic acid derivatives, such as isoferulic, cinnamic, 2-coumaric, 3-coumaric, 4-coumaric, and caffeic acids, but did not accept ferulic acid and sinapic acid (Suppl. Figs. S8 and S10). The acceptance of substituted cinnamic acid derivatives (except sinapic acid) might be based on size exclusion. As described by Schneider et al. (2003), ferulic acid was accepted when the amino acids methionine and lysine (fourth and fifth amino acid of the 4CL substrate specificity code) were exchanged with amino acids with shorter side chains. Both Aa4HBCL and Aa4CL shared the amino acids proline and methionine at these positions. While Aa4CL accepted both ferulic and isoferulic acid, Aa4HBCL lacked affinity for ferulic acid but activated isoferulic acid. Thus, there might still be some unsolved questions concerning the 4CL substrate specificity code. Nonetheless, the specific acitvities of Aa4HBCL using cinnamic acid derivatives were about tenfold lower compared to Aa4CL. For both enzymes, the formation of AMP could be observed even for the not-converted substrates. Thus, the enzymes were able to cleave ATP to AMP and pyrophosphate even without any suitable acceptor substrate.

The *K*_m_ value for 4HBA was rather high at 664.2 ± 1.5 µM. This value can only be compared to badA from *Rhodopseudomonas palustris* (158 µM) (Thornburg et al. 2015) since there are no available kinetic parameters for respective plant enzymes. The benzoate-activating enzyme from *C. breweri* (Beuerle and Pichersky 2002) displayed a *K*_m_ value of 95 µM for ATP. With 1.2 ± 0.1 mM Aa4HBCL had a much lower affinity. Also, the *K*_m_ value of 13 µM from *C. breweri* for CoA is much lower than the *K*_m_ value of Aa4HBCL with 247.8 ± 19.9 µM (Table 2).

The importance of the phenylpropanoid pathway in the hornwort *Anthoceros agrestis* is depicted in the presence of at least two genes encoding phenylalanine ammonia-lyase (Pezeshki et al. in progress) and one for cinnamic acid 4-hydroxylase (CYP73A260; Wohl and Petersen 2020) as well as four putative CoA ligase genes, two of which have been described here. The strongly different substrate preferences of the two characterized CoA ligases, one preferring (hydroxy)cinnamic acids (Aa4CL, GenBank MN922305), the other (hydroxy)benzoic acids (Aa4HBCL, GenBank MN922306), are reflected in the spectrum of phenolic compounds identified in *Anthoceros agrestis* by Trennheuser (1992) as well as Takeda et al. (1990) and Vogelsang et al. (2006). Besides free acids, various esters and amides of 4-hydroxybenzoic, protocatechuic, vanillic, 4-coumaric, caffeic and isoferulic acids were isolated while ferulic acid esters were not found. The substrate spectra of the two CoA ligases described in this work perfectly fit to this compound spectrum. The formation of these esters and amides requires the presence of (hydroxy)benzoyl- and hydroxycinnamoyltransferases which are currently under investigation in our laboratory.

## Conclusion

A 4CL and a 4HBCL from the hornwort *Anthoceros agrestis* were amplified and heterologously expressed in *E. coli.* This resulted in catalytically active 4CL and 4HBCL proteins which were characterized biochemically. While Aa4CL only accepted hydroxycinnamic acids, Aa4HBCL activated benzoic acid derivatives and some hydroxycinnamic acids.

### Author contribution statement

JW and MP conceived and designed the research. JW conducted the experiments, and JW and MP analyzed the data and wrote the manuscript. Both authors read and approved the manuscript.

## Electronic supplementary material

Below is the link to the electronic supplementary material.

Supplementary file1 (PDF 2587 kb)
