# Supplementary material for: Phenolic metabolism in the hornwort Anthoceros agrestis: 4-coumarate CoA ligase and 4-hydroxybenzoate CoA ligase
Source: Plant Cell Rep. 2020 May 13;39(9):1129–41. doi: 10.1007/s00299-020-02552-w (PMC7419483; doi:10.1007/s00299-020-02552-w)
Supplement: Supplementary file 1 — Supplementary file1 (PDF 2587 kb) [file 299_2020_2552_MOESM1_ESM.pdf]

Supplemental Material

Julia Wohl, Maike Petersen

Phenolic metabolism in the hornwort *Anthoceros agrestis*: 4-Coumarate CoA Ligase and 4-hydroxybenzoate CoA ligase

Institut für Pharmazeutische Biologie und Biotechnologie, Philipps-Universität Marburg, Robert-Koch-Str. 4, D-35037 Marburg, Germany

Suppl. Table S1 List of PCR primers: binding sequence underlined and restriction sites marked red

Primer name	Sequence
AaAp6378_f	TTCCGCTCCAACTTGCCCG
Aa Ap6378_r	ATCCTCCAGCTCCTTCCCC
Aa Ap6378_5´R	CTCGACACCACCTCCACCTCGCTGTAG
Aa Ap6378_3´R	GTGATGCTGTGCGGGCTGCGTGCT
Aa Ap6378_fl1_f	TATCTGCAGATGGCGCCGATTCTTGCACCTCC
Aa Ap6378_fl2_f	TATCTGCAGATGCCTGCGGAGATGGAGGCC
Aa Ap6378_fl_r	TATAAGCTTCTACACCCTGCTCCTCAGCTCC
Aa35279_f	TCTTCGGGGACAACAGGACTG
Aa35279_r	AACCTGGAACCCTTTGTACTTGATG
Aa35279_ 5´R	CCGTTGTGCCGTAAATGTGGAAGAAGGGC
Aa35279_3´R	GGAAAGTCCCTGCCTGCAAATCGTG
Aa35279_fl_f	TATCTCGAGATATGGCCTCTCTCTCCGAGCC
Aa35279_fl_r	TATAAGCTTTCAAATTAGTTGGTTCTCAGTCTTCTTGAG

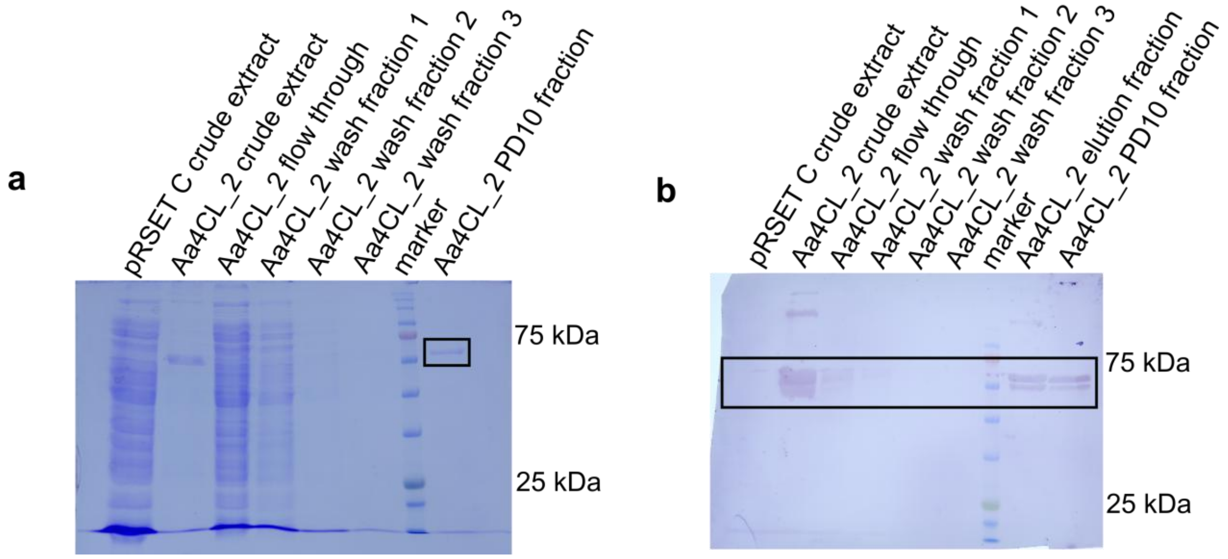

Suppl. Fig. S1 SDS-Page and Western blot of Aa4CL_2 heterologously expressed in *E. coli* SoluBL21. (a) Coomassie stained SDS-PAGE gel of heterologously expressed Aa4CL_2 with *N*-terminally attached 6xHis-tag before and after purification by metal chelate chromatography and equally treated crude protein extract from *E. coli* SoluBL21 carrying the empty vector pRSET C. (b) Western blot after detection using anti-6x-His-tag monoclonal mouse antibody and goat anti-mouse antibody conjugated to alkaline phosphatase and NBT/BCIP colour reaction

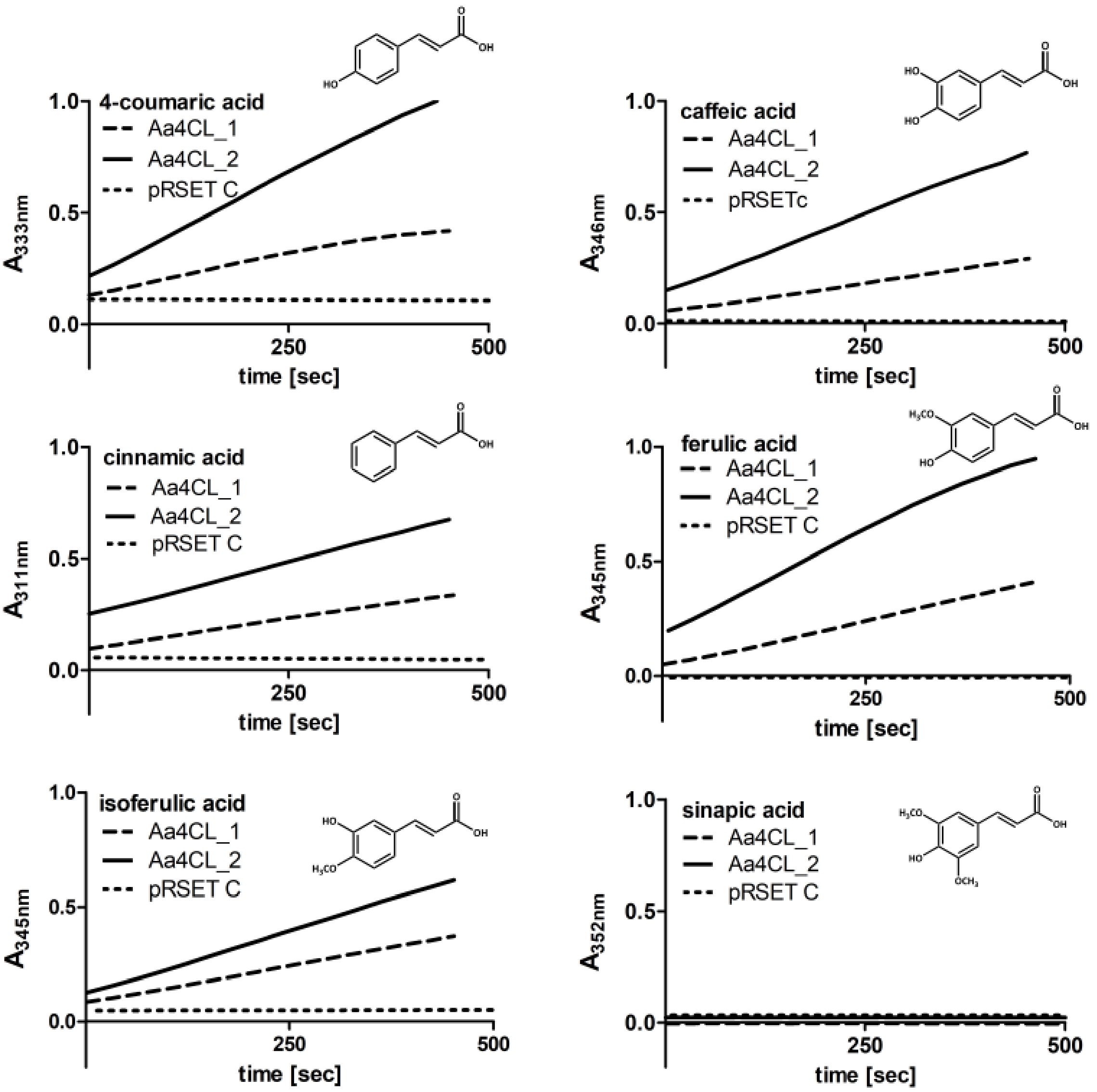

Suppl. Fig. S2 Activity of purified Aa4CL_1 and Aa4CL_2 (3 µg protein) heterologously expressed in *E. coli* with different hydroxycinnamic acid substrates (500 µM). Crude protein extract (60 µg protein) from transformed *E. coli* with the empty vector pRSET C served as a negative control

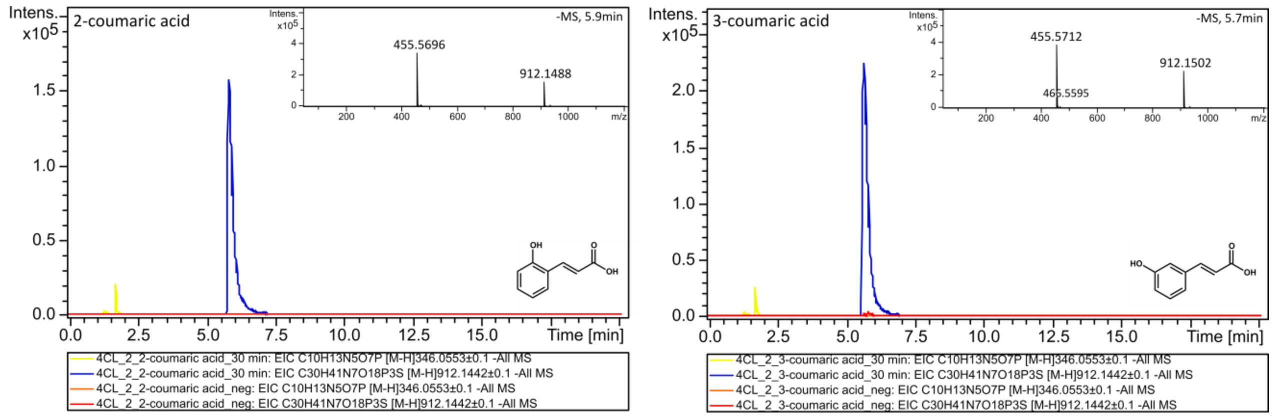

Suppl. Fig. S3 LC-MS analysis of CoA esters formed by Aa4CL with 2- and 3-coumaric acid. Purified Aa4CL_2 (8 µg protein) was incubated with 500 µM substrate, 1.25 mM ATP and 1 mM CoA for 30 min at 40 °C. Heat-denatured protein (10 min 95 °C) was used as a negative control. The chromatograms show the EIC of the expected products AMP (m/z 346.0553 ± 0.1) in yellow for Aa4CL_2 and orange for the negative control. The corresponding CoA-ester is displayed in blue for Aa4CL_2 or red for the negative control. The exact mass of the resulting CoA-ester is shown in each chromatogram. For all produced CoA-esters we observed the [M-H] pseudo molecular ion (m/z 912.15) as well as the doubly charged molecular ion (m/z 455.57) [M/2]-H

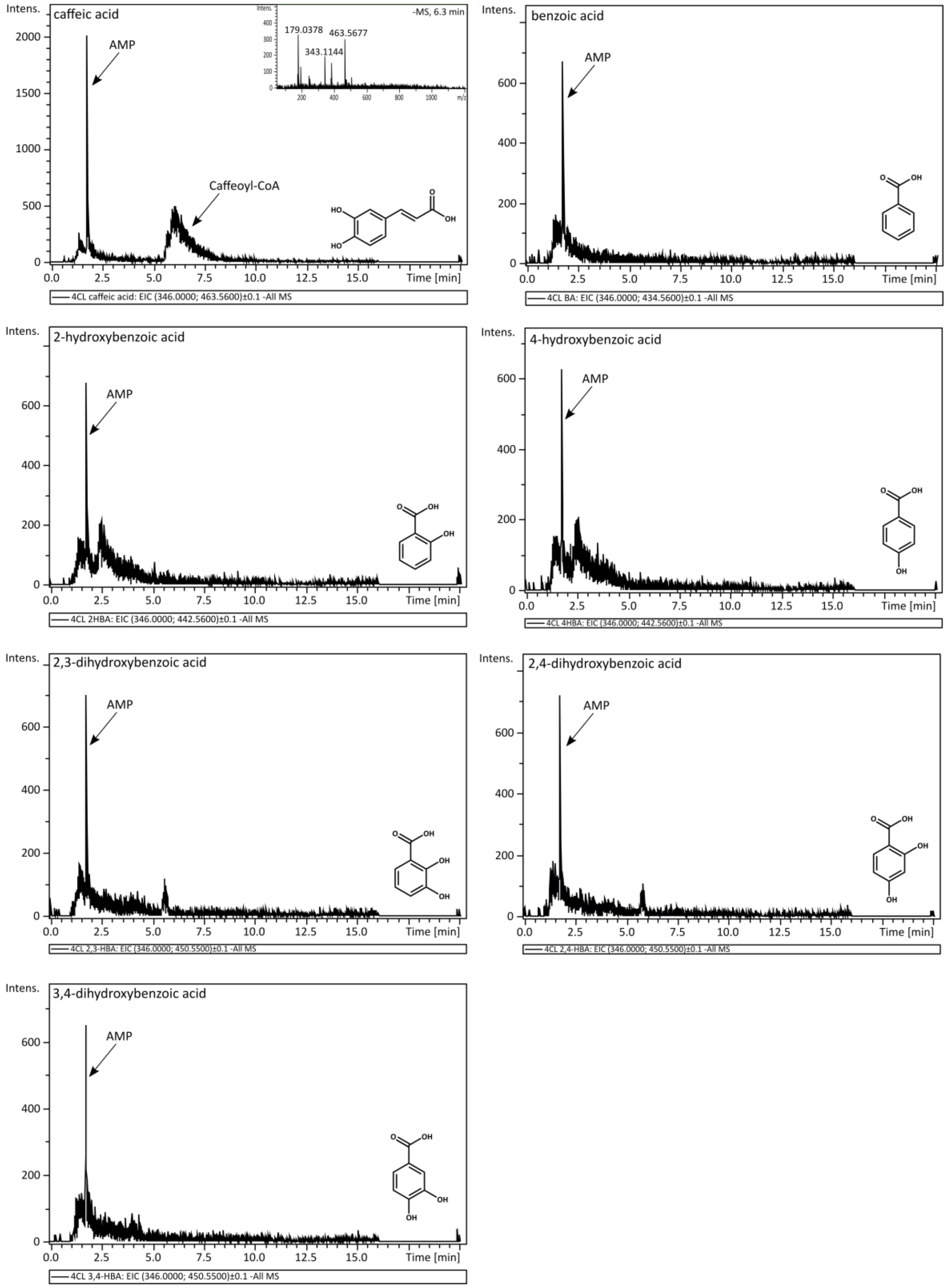

Suppl. Fig. S4 LC-MS analysis of CoA esters formed by Aa4CL with various (hydroxy)benzoic acids and caffeic acid. Purified Aa4CL_2 (7.5 µg protein) was incubated with 500 µM caffeic, benzoic, 2-hydroxybenzoic, 4-hydroxybenzoic, 2,3-dihydroxybenzoic, 2,4-dihydroxybenzoic or 3,4-dihydroxybenzoic acids for 1 h at 35 °C. The chromatograms show the EIC of the expected products AMP (m/z 346 ± 0.1) and the corresponding CoA-ester (doubly charged molecular ion [M/2]-H) of caffeic acid (m/z 463.56 ± 0.1), benzoic acid (m/z 434.56 ± 0.1), monohydroxylated benzoic acids (m/z 442.56 ± 0.1) or dihydroxylated benzoic acids (m/z 450.55 ± 0.1)

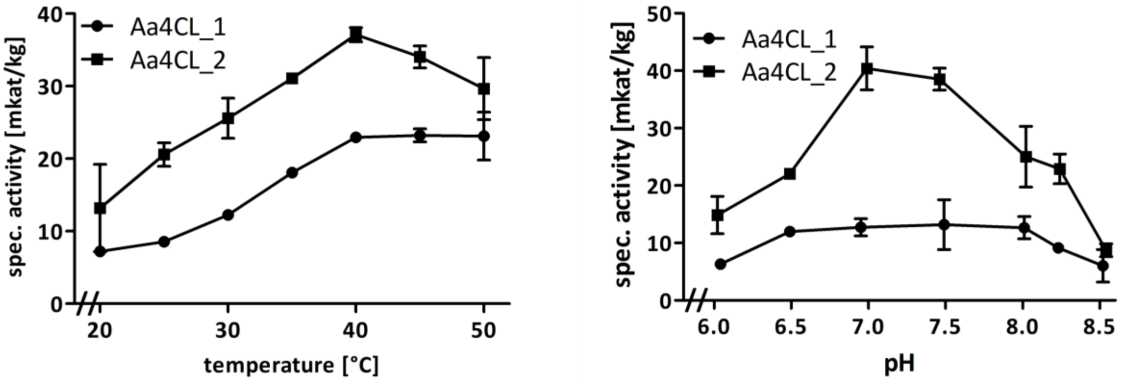

Suppl. Fig. S5 Temperature (left) and pH-optimum (right) of Aa4CL_1 and Aa4CL_2 (mean ± SD, n=7)

Michaelis-Menten **Hanes-Woolf**

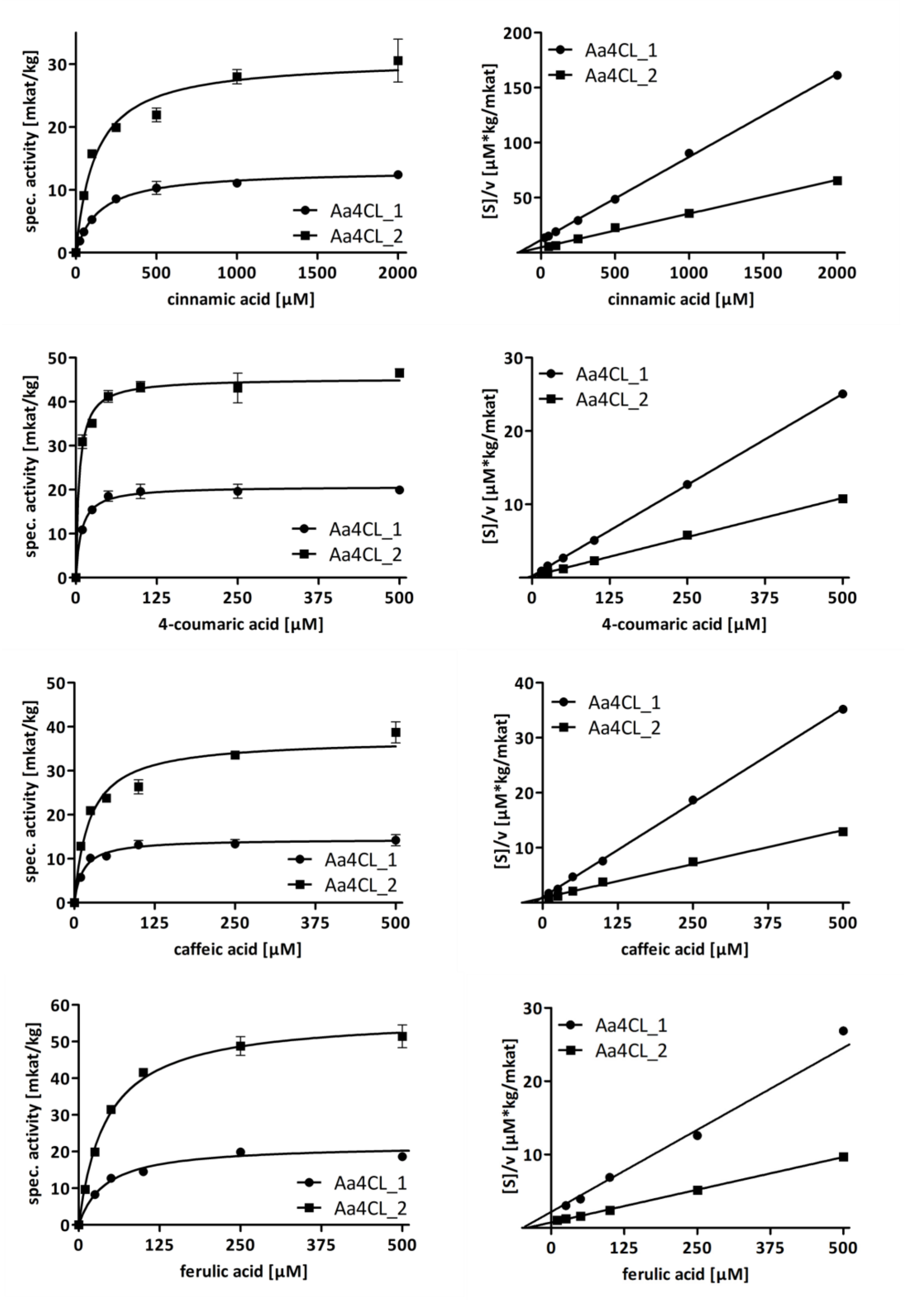

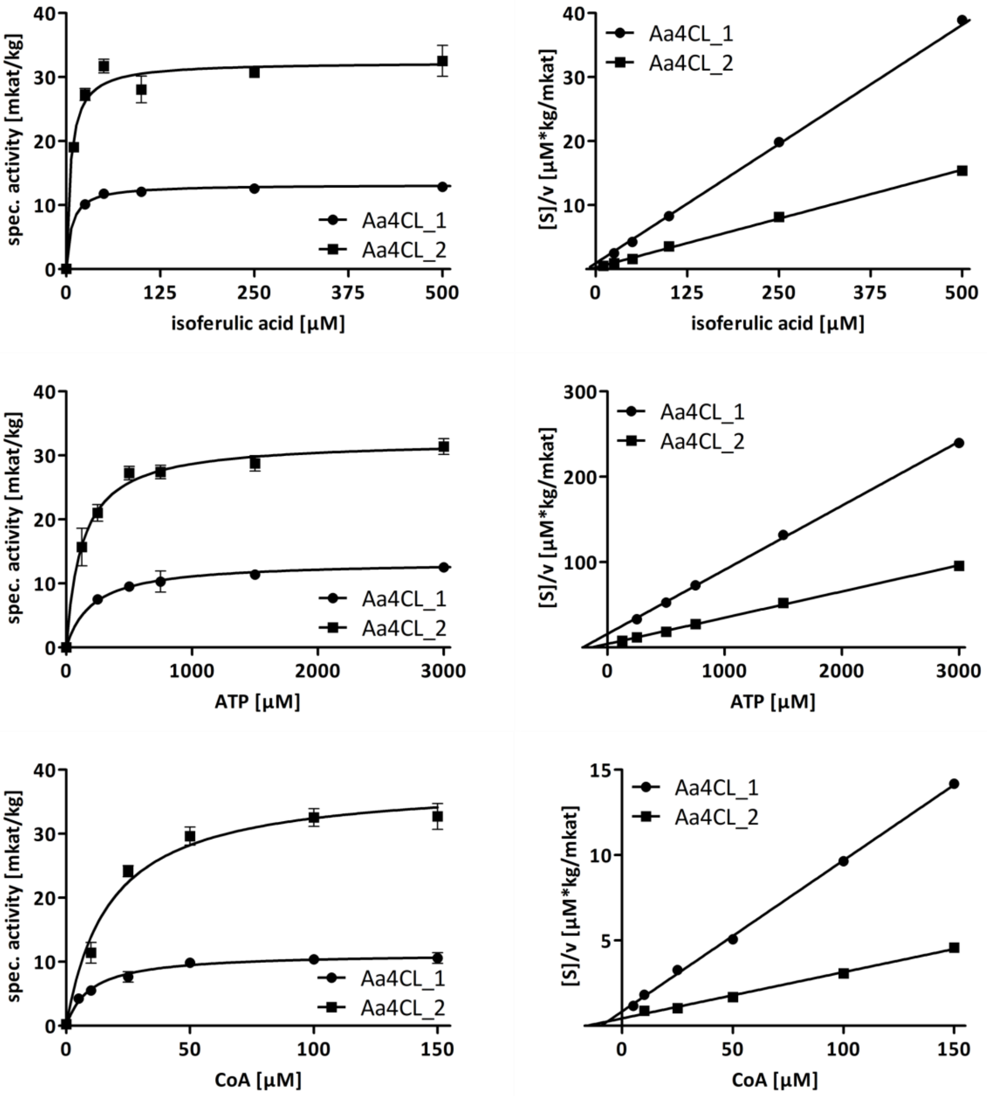

Suppl. Fig. S6 Substrate saturation curves of reactions of Aa4CL_1 and Aa4CL_2 for the determination of K_m-values (mean ± SD, n = 9). Hydroxycinnamic acids were incubated with 2.5 mM ATP and 100 µM CoA. For the determination of K_m-values for ATP 100 µM CoA and for CoA 2.5 mM ATP were used together with 500 µM caffeic acid. Michaelis-Menten diagrams are shown on the left and Hanes-Woolf diagrams on the right

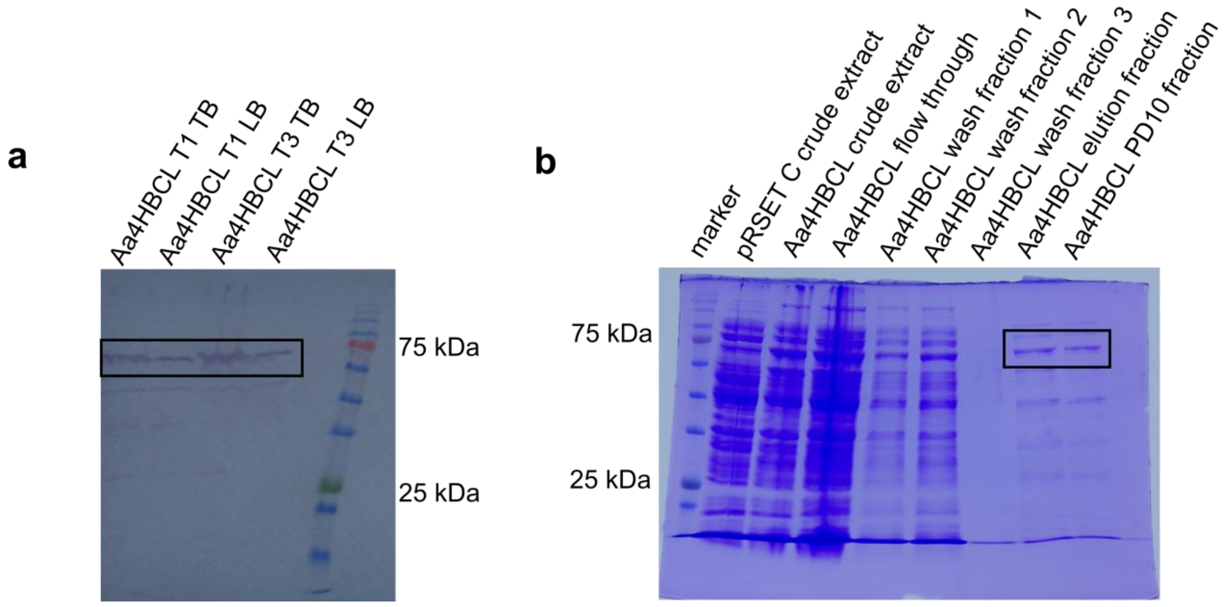

Suppl. Fig. S7 Western blot and SDS-Page of Aa4HBCL heterologously expressed in *E. coli* SoluBL21. (a) Western blot analysis of His-tagged Aa4HBCL expressed in two different *E. coli* SoluBL21 transformants (T1 or T3) in either TB or LB media. (b) Coomassie stained SDS-PAGE gel of heterologously expressed Aa4HBCL with *N*-terminally attached 6xHis-tag before and after purification by metal chelate chromatography and equally treated crude protein extract from *E. coli* carrying the empty vector pRSET C

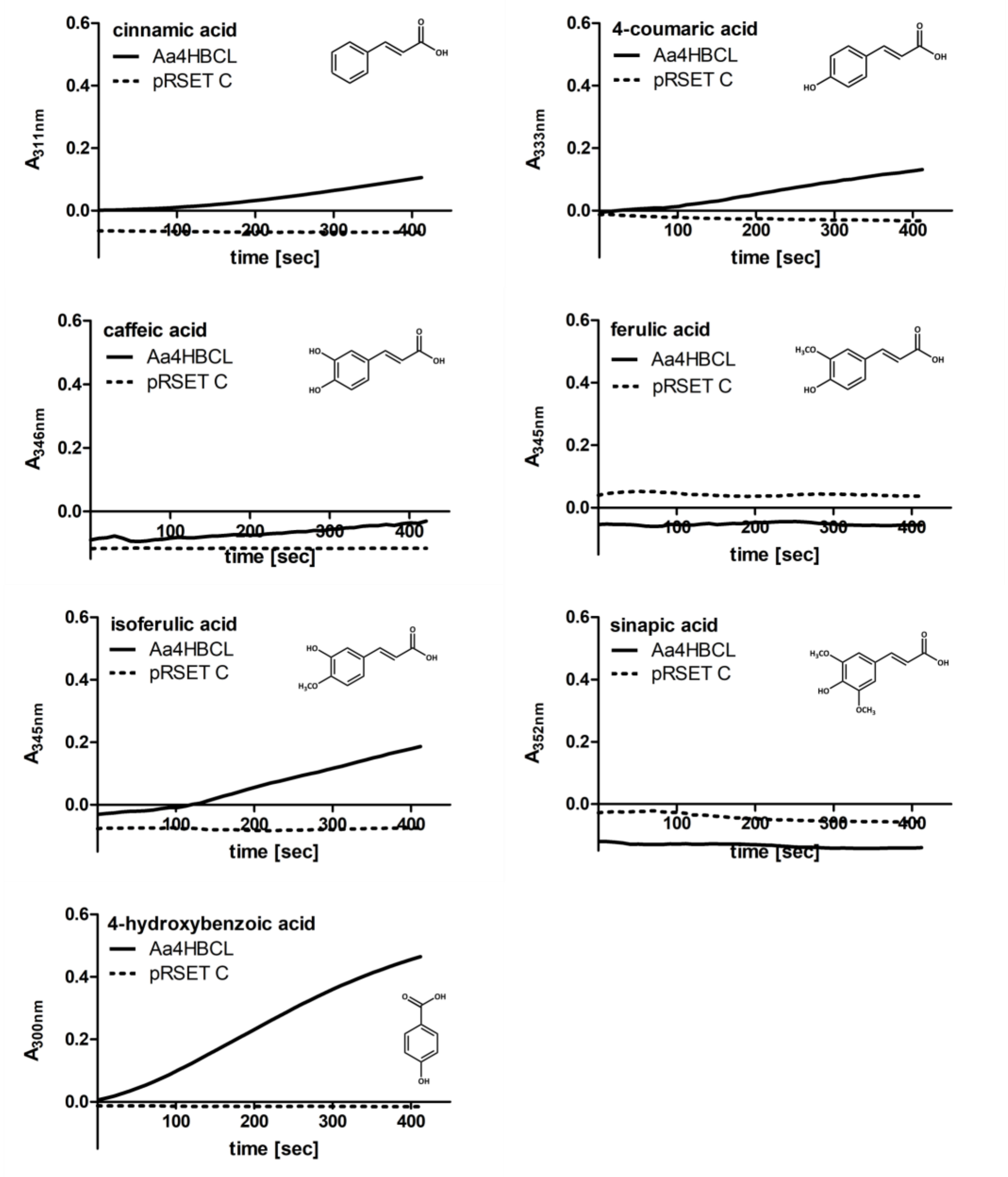

Suppl. Fig. S8 Activity of heterologously expressed purified Aa4HBCL (32 µg protein) with 500 µM 4-hydroxybenzoic, cinnamic, 4-coumaric, caffeic, ferulic, isoferulic or sinapic acids. Crude protein extract (60 µg protein) from transformed *E. coli* with the empty vector pRSET C served as a negative control

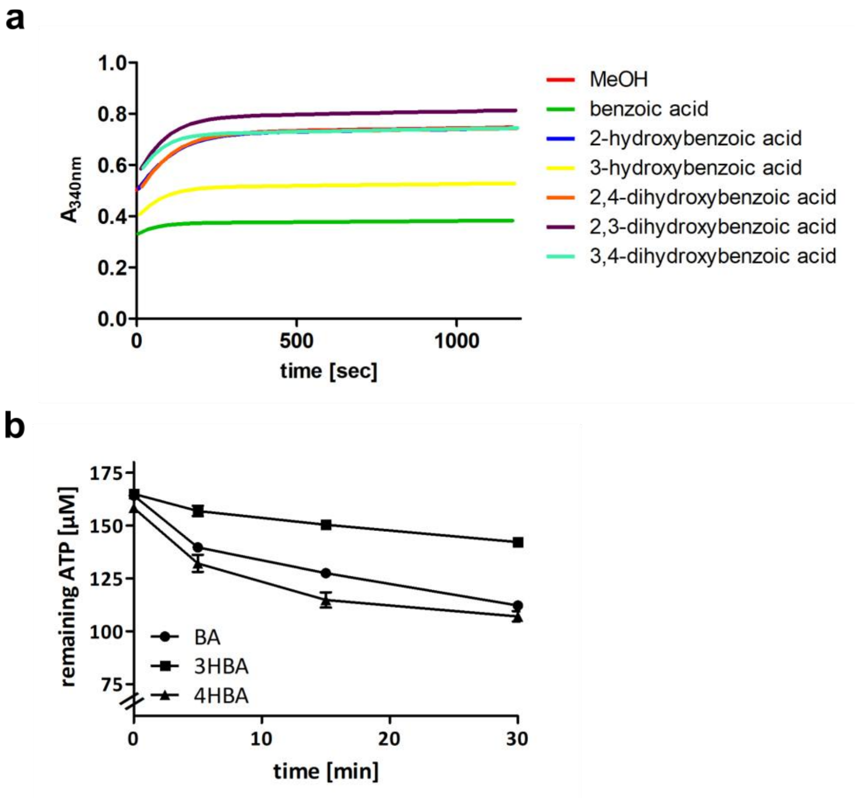

Suppl. Fig. S9 Assays of Aa4HBCL (14 µg protein) with different benzoic acid derivatives measured by the indirect method with hexokinase and glucose-6-phosphate dehydrogenase. (a) Aa4HBCL incubated with 500 µM benzoic acid, monohydroxylated or dihydroxylated benzoic acids and 500 µM ATP and 500 µM CoA for 1 h at 40 °C before addition of reaction mixtures containing glucose, hexokinase, NADP and glucose-6-phosphate dehydrogenase. MeOH instead of substrate was used as a negative control. (b) Decreasing ATP concentration of Aa4HBCL assays incubated with 500 µM benzoic acid (BA), 3-hydroxybenzoic acid (3HBA) or 4-hydroxybenzoic acid (4HBA) and 500 µM ATP and 500 µM CoA after 0, 5, 15 and 30 min of incubation at 45 °C before addition of reaction mixtures containing glucose, hexokinase, NADP and glucose-6-phosphate dehydrogenase (mean ± SD, n = 3). A calibration curve of different ATP concentrations was used to calculate the remaining ATP concentration in the assays

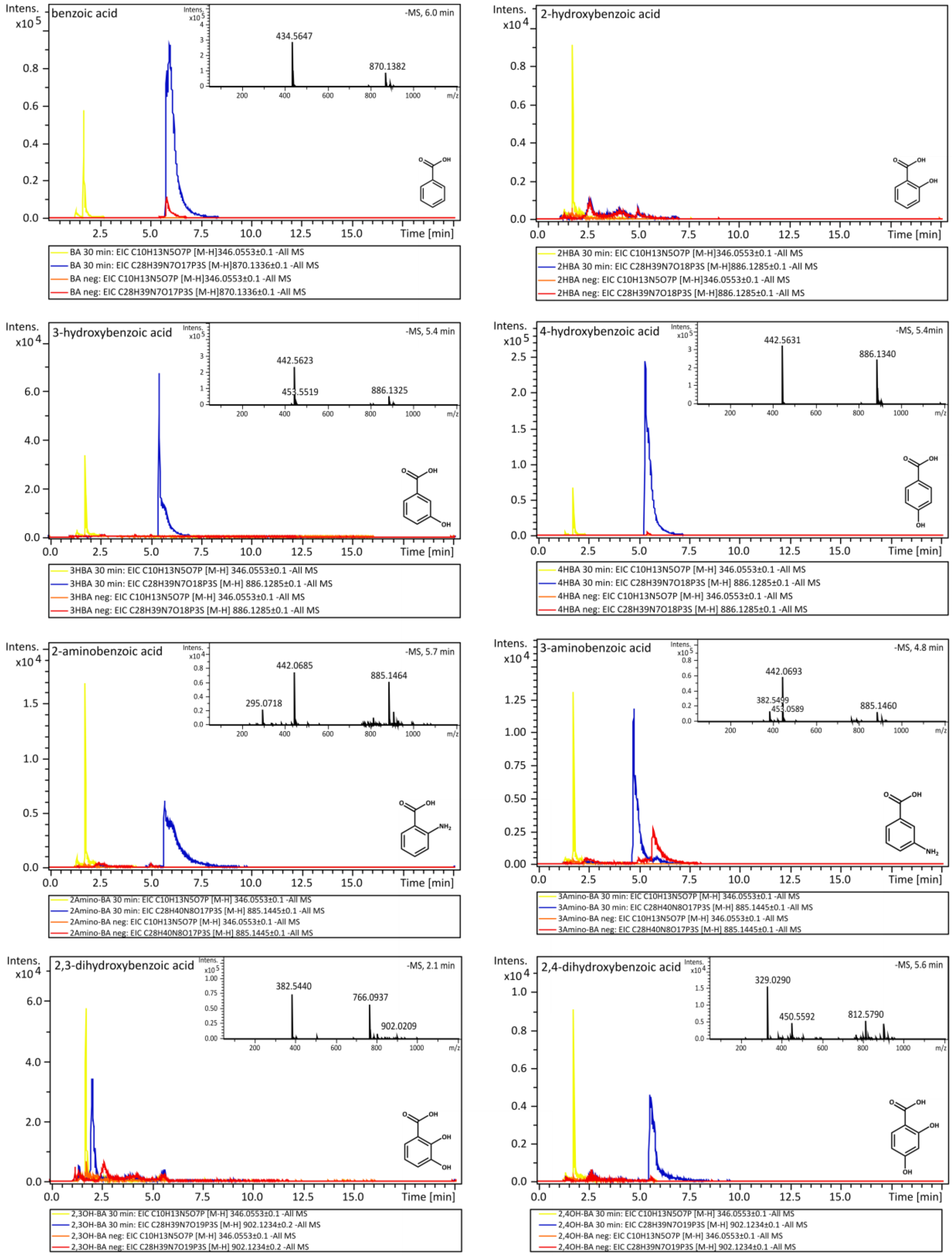

benzoic acid
-MS, 6.0 min
434.5647
870.1382
BA 30 min: EIC C10H13N5O7P [M-H]346.0553±0.1 -All MS
BA 30 min: EIC C28H39N7O17P3S [M-H]870.1336±0.1 -All MS
BA neg: EIC C10H13N5O7P [M-H]346.0553±0.1 -All MS
BA neg: EIC C28H39N7O17P3S [M-H]870.1336±0.1 -All MS
2-hydroxybenzoic acid
2HBA 30 min: EIC C10H13N5O7P [M-H]346.0553±0.1 -All MS
2HBA 30 min: EIC C28H39N7O18P3S [M-H]886.1285±0.1 -All MS
2HBA neg: EIC C10H13N5O7P [M-H]346.0553±0.1 -All MS
2HBA neg: EIC C28H39N7O18P3S [M-H]886.1285±0.1 -All MS
3-hydroxybenzoic acid
-MS, 5.4 min
442.5623
886.1325
3HBA 30 min: EIC C10H13N5O7P [M-H] 346.0553±0.1 -All MS
3HBA 30 min: EIC C28H39N7O18P3S [M-H] 886.1285±0.1 -All MS
3HBA neg: EIC C10H13N5O7P [M-H] 346.0553±0.1 -All MS
3HBA neg: EIC C28H39N7O18P3S [M-H] 886.1285±0.1 -All MS
4-hydroxybenzoic acid
-MS, 5.4min
442.5631
886.1340
4HBA 30 min: EIC C10H13N5O7P [M-H] 346.0553±0.1 -All MS
4HBA 30 min: EIC C28H39N7O18P3S [M-H] 886.1285±0.1 -All MS
4HBA neg: EIC C10H13N5O7P [M-H] 346.0553±0.1 -All MS
4HBA neg: EIC C28H39N7O18P3S [M-H] 886.1285±0.1 -All MS
2-aminobenzoic acid
-MS, 5.7 min
442.0685
885.1464
295.0718
2Amino-BA 30 min: EIC C10H13N5O7P [M-H] 346.0553±0.1 -All MS
2Amino-BA 30 min: EIC C28H40N8O17P3S [M-H] 885.1445±0.1 -All MS
2Amino-BA neg: EIC C10H13N5O7P [M-H] 346.0553±0.1 -All MS
2Amino-BA neg: EIC C28H40N8O17P3S [M-H] 885.1445±0.1 -All MS
3-aminobenzoic acid
-MS, 4.8 min
442.0693
885.1460
3Amino-BA 30 min: EIC C10H13N5O7P [M-H] 346.0553±0.1 -All MS
3Amino-BA 30 min: EIC C28H40N8O17P3S [M-H] 885.1445±0.1 -All MS
3Amino-BA neg: EIC C10H13N5O7P [M-H] 346.0553±0.1 -All MS
3Amino-BA neg: EIC C28H40N8O17P3S [M-H] 885.1445±0.1 -All MS
2,3-dihydroxybenzoic acid
-MS, 2.1 min
382.5440
766.0937
902.0209
2,3OH-BA 30 min: EIC C10H13N5O7P [M-H] 346.0553±0.1 -All MS
2,3OH-BA 30 min: EIC C28H39N7O19P3S [M-H] 902.1234±0.2 -All MS
2,3OH-BA neg: EIC C10H13N5O7P [M-H] 346.0553±0.1 -All MS
2,3OH-BA neg: EIC C28H39N7O19P3S [M-H] 902.1234±0.2 -All MS
2,4-dihydroxybenzoic acid
-MS, 5.6 min
329.0290
450.5592
812.5790
2,4OH-BA 30 min: EIC C10H13N5O7P [M-H] 346.0553±0.1 -All MS
2,4OH-BA 30 min: EIC C28H39N7O19P3S [M-H] 902.1234±0.1 -All MS
2,4OH-BA neg: EIC C10H13N5O7P [M-H] 346.0553±0.1 -All MS
2,4OH-BA neg: EIC C28H39N7O19P3S [M-H] 902.1234±0.1 -All MS
Time [min]

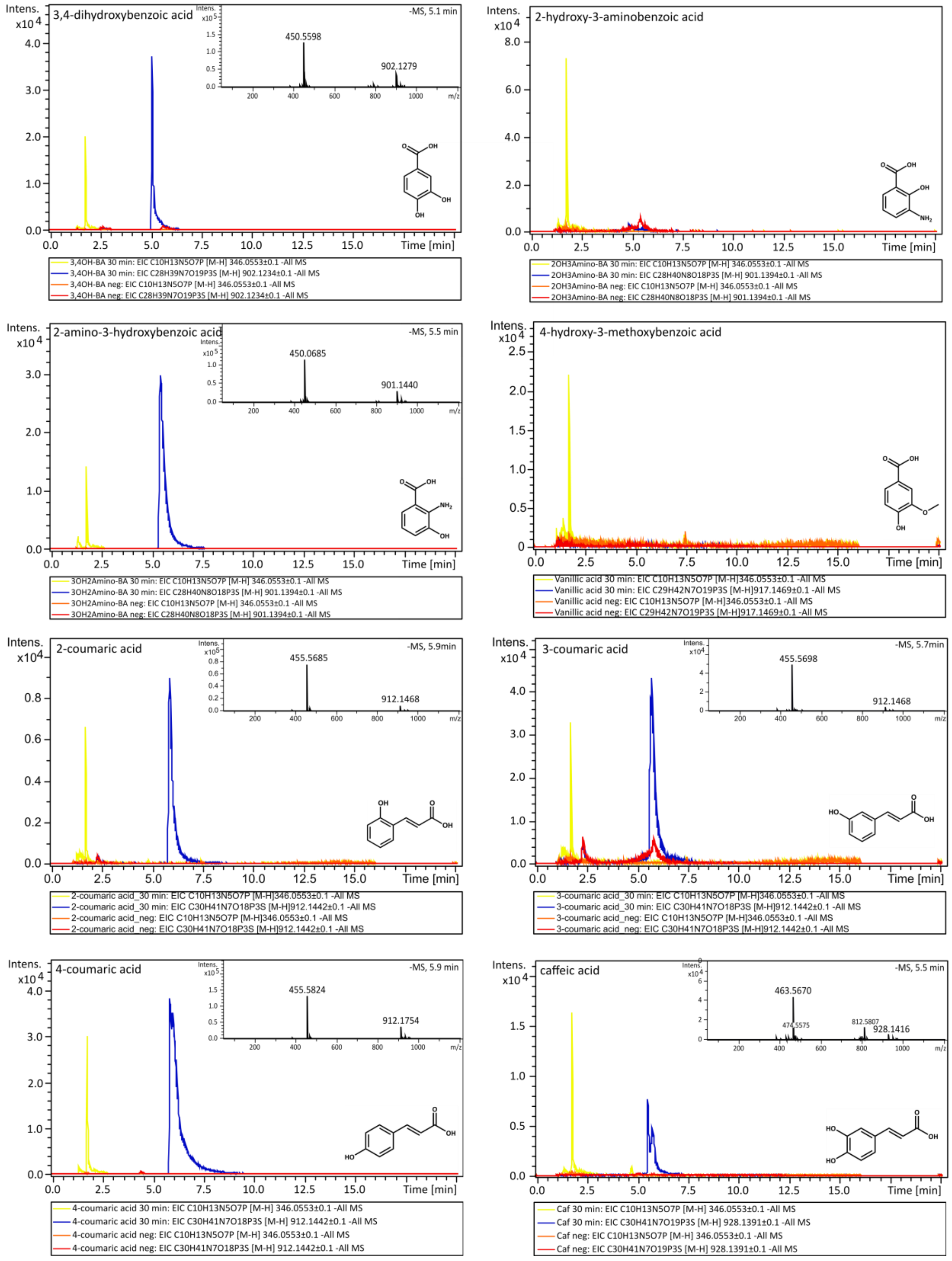

Suppl. Fig. S10 LC-MS analysis of reaction products of Aa4HBCL with different (hydroxy)benzoic and (hydroxy)cinnamic acids. Purified Aa4HBCL (28 µg protein) was incubated with 500 µM substrate, 1.25 mM ATP and 1 mM CoA for 30 min at 40 °C. Heat-denatured protein (10 min 95 °C) was used as a negative control. The chromatograms show the EIC of the expected products AMP (m/z 346.0553 ± 0.1) in yellow for Aa4HBCL and

orange for the negative control. The corresponding CoA-ester is displayed in blue for Aa4HBCL or red for the negative control. The exact mass of the resulting CoA-ester is shown in each chromatogram. For all produced CoA-esters we observed the [M-H] pseudo molecular ion as well as the doubly charged molecular ion [M/2]-H

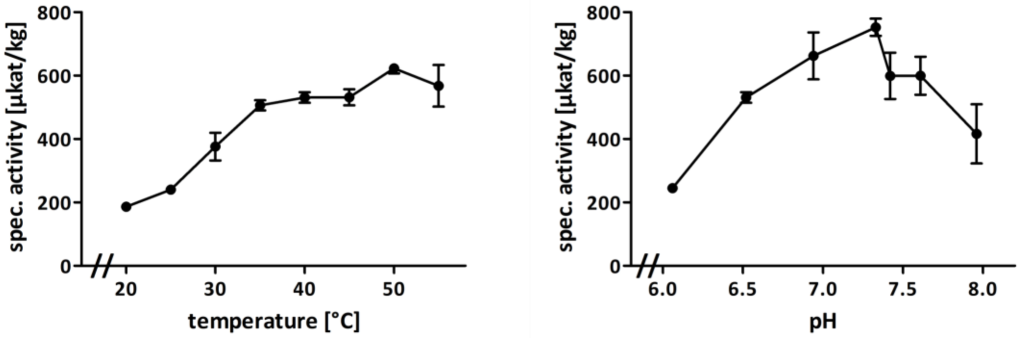

Suppl. Fig. S11 Temperature (left) and pH-optimum (right) of Aa4HBCL (mean ± SD, n = 3)

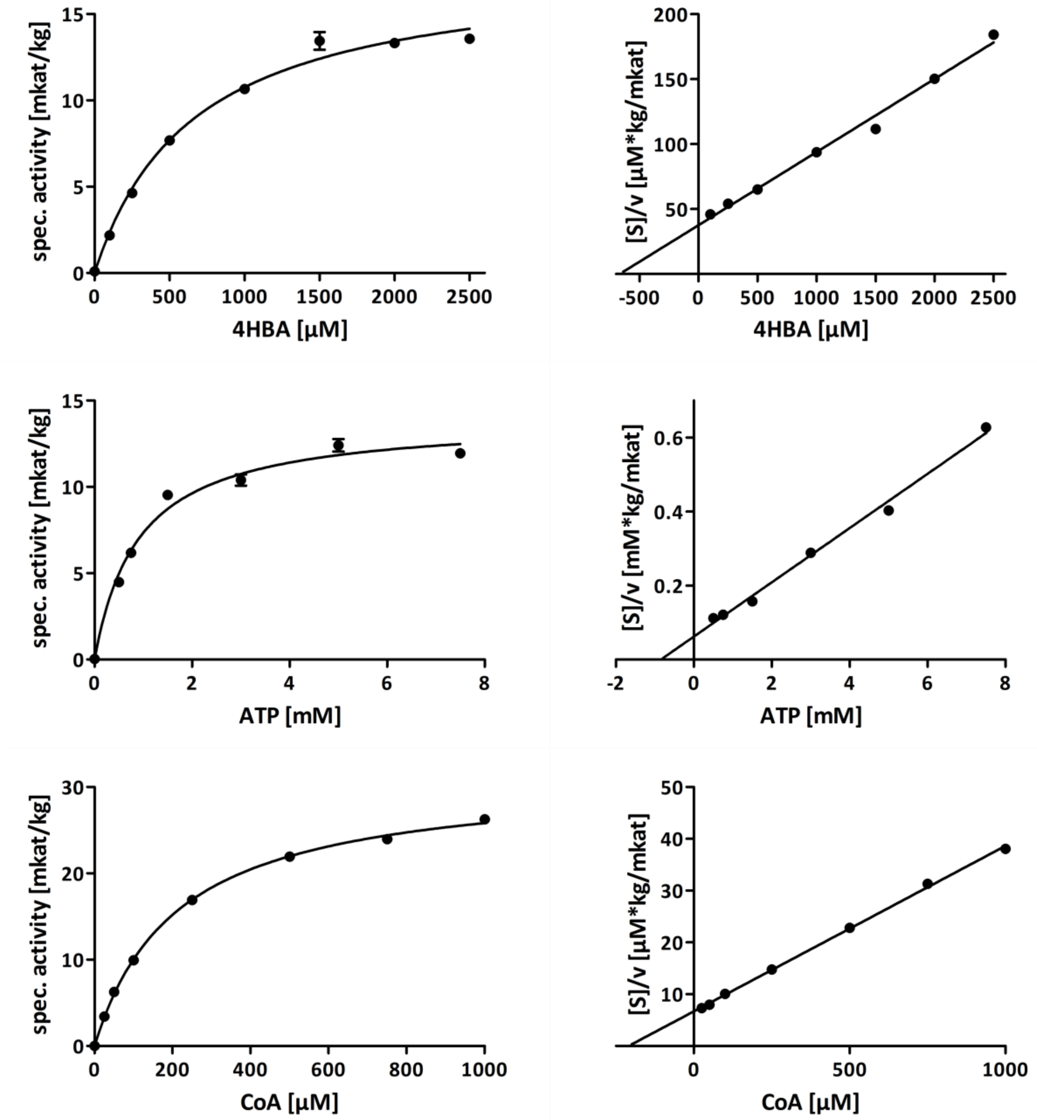

Suppl. Fig. S12 Dependence of Aa4HBCL on 4-hydroxybenzoic acid (4HBA), ATP and CoA for the determination of K_m-values (mean ± SD, n = 9). Michaelis-Menten diagrams are displayed on the left and Hanes-Wolf diagrams on the right. 4HBA was incubated with 4 mM ATP and 750 µM CoA. Determination of K_m-values for ATP were measured with 2.5 mM 4HBA and 750 µM CoA. Different CoA concentrations were incubated with 2.5 mM 4HBA and 5 mM ATP
